# Hand Hygiene – Evaluation of Three Disinfectant Hand Sanitizers in a Community Setting

**DOI:** 10.1371/journal.pone.0111969

**Published:** 2014-11-07

**Authors:** Rita Babeluk, Sabrina Jutz, Sarah Mertlitz, Johannes Matiasek, Christoph Klaus

**Affiliations:** 1 Department of Surgery, Medical University of Vienna, Vienna, Austria; 2 Department of Biotechnology, University of Applied Science Campus Vienna, Vienna, Austria; 3 Department of Plastic, Aesthetic and Reconstructive Surgery, Wilhelminen Hospital Vienna, Vienna, Austria; 4 International Scientific Affairs, Schülke & Mayr GmbH, Vienna, Austria; The University of Tokyo, Japan

## Abstract

Hand hygiene is acknowledged as the single most important measure to prevent nosocomial infections in the healthcare setting. Similarly, in non-clinical settings, hand hygiene is recognised as a key element in helping prevent the spread of infectious diseases. The aim of this study was to evaluate the efficacy of three different disinfectant hand sanitizers in reducing the burden of bacterial hand contamination in 60 healthy volunteers in a community setting, both before and after education about the correct use of hand sanitizers. The study is the first to evaluate the efficacy and ease of use of different formulations of hand rubs used by the general population. The products tested were: Sterillium (perfumed, liquid), desderman pure gel (odorless, gel) and Lavit (perfumed, spray). Sterillium and desderman are EN1500 (hygienic hand rub) certified products (available in pharmacy) and Lavit is non EN1500 certified and available in supermarkets. The two EN1500 certified products were found to be significantly superior in terms of reducing bacterial load. desderman pure gel, Sterillium and Lavit reduced the bacterial count to 6.4%, 8.2% and 28.0% respectively. After education in the correct use of each hand rub, the bacterial load was reduced even further, demonstrating the value of education in improving hand hygiene. Information about the testers' perceptions of the three sanitizers, together with their expectations of a hand sanitizer was obtained through a questionnaire. Efficacy, followed by skin compatibility were found to be the two most important attributes of a hand disinfectant in our target group.

## Introduction

The word hygiene derives from the ancient Greek goddess Hygeia, the goddess of healing. [1] Today, hygiene is associated with disease prevention and health promotion. The importance of hygiene is universally recognised and evidence based. [2] Physical contact between people and between people and objects is a key vehicle for the transmission of pathogens. Therefore, effective hand hygiene is a key intervention in disease prevention. [3] It is an integral procedure in the healthcare environment, with healthcare workers receiving regular training about hand hygiene procedures. [4], [5], [6], [7], [8], [9], [10], [11].

In the community, outside of the healthcare environment, studies have reported an association between improvements in hand hygiene and reductions in rates of infectious diseases. [3] It is estimated that simple hand washing could save one million lives a year [12], [13] and many public health campaigns worldwide have addressed “hand hygiene” with varying success. [14], [15], [16].

However, studies show that after hand washing, as many as 80% of individuals retain some pathogenic bacteria on their hands. [17] Hand washing with soap removes the body's own fatty acids from the skin, which may result in cracked skin that provides an entry portal for pathogens [18], [19] In contrast, high-quality hand disinfectants contain additional skin care products, like emollients. [20] They also do not require the use of water, which makes the application easy and uncomplicated.

Adherence to hand hygiene practices amongst healthcare professionals has been regularly audited and investigated. [21] Whereas to our knowledge, hand hygiene practices amongst the general population have rarely been examined. Furthermore, the efficacy of different formulations of hand disinfectants on bacterial load reduction in non-healthcare settings has not been previously investigated.

## Material and Methods

Sixty undergraduate students of the *University of Applied Sciences, Campus Vienna* took part in this study in April 2013. The cohort was chosen to guarantee that the same volunteers could participate in both parts of the study. The students had no prior training in hand hygiene and were therefore believed to be representative of the general (non-healthcare) population.

**Figure 1:**
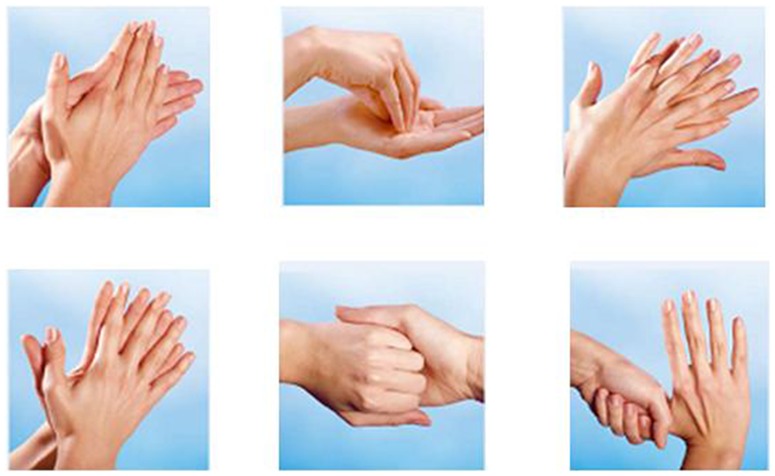
Hand disinfection steps according to EN1500.

European Norm (EN) 1500 is utilised in Europe for in vivo testing of hygienic hand rubs that are designed to reduce the level of transient flora on the hands. [22] EN 1500 requires 18–22 test volunteers and the usage of 3 ml of the hand rub agent for 30 seconds in a defined process to examine the efficacy of a hand disinfectant. These guidelines were followed in the study. (Fig. 1).

**Figure 2:**
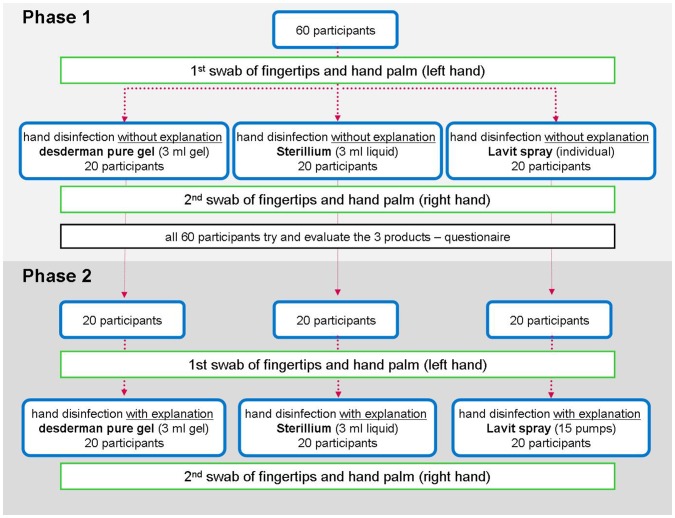
Study design.

Three products were tested, and each was tested by the same 20 people in two phases within one week. (Fig. 2) The three hand sanitizers selected for testing were: 3 ml Sterillium (45.0 g propan-2-ol, 30.0 g propan-1-ol, 0.2 g mecetroniumetilsulfate/100.0 g; perfumed, liquid), 3 ml desderman pure gel (78.2 g ethanol (96%), 0.1 g biphenyl-2-ol/100.0 g; odorless, gel) and Lavit (alcohol based, not specified; perfumed, spray). Sterillium and desderman pure gel are EN1500 certified products (available in pharmacies), whereas Lavit is not an EN1500 certified product (available in supermarkets). All three products are easily available to consumers, but have different characteristics. desderman pure gel costs 1.96 €/100 ml, Sterillium 2.15 €/100 ml, (according to 2013 market prices in Austrian pharmacies) and Lavit 3.99 €/15 ml; 26.6 €/100 ml.

The 60 volunteers were briefed to attend the two testing sessions at the University, using public transport. They were also asked to contaminate their hands by touching typical everyday surfaces (i.e. hand rails, door handles, vending machines, money) with both hands prior to testing.

On arrival at the University laboratory, a swab of each subject's left palm and finger tips was taken and cultured on agar plates to determine a maximum spectrum of microbes present (HYCON Contact Slides TC; Merck Millipore). This provided the base line for the testing. The hand preference of the participants was not evaluated as it is known that 90% of the population is right handed. [23] However, the ‘less likely to be contaminated’ left hand was consistently tested for bacterial load prior to hand disinfection, to ensure the accuracy of results.

The volunteers were randomized into three groups of 20, each group was allocated one of the three hand sanitizers: desderman pure gel, Sterillium or Lavit. Gender and age distribution were not significantly different between the three groups. The subjects had no previous training in hand disinfection and were asked to use the supplied product without being given specific instructions, apart from reading the manufacturers' labels. After applying the hand rub, swabs were taken from each subject's right palm and fingertips; and cultured on agar plates (HYCON Contact Slides TC; Merck Millipore) at 35°C for 24 hours to determine a maximum spectrum of microbes present. The media is able to determine the total bacterial bio burden including Gram positive as well as Gram negative forms.

**Figure 3:**
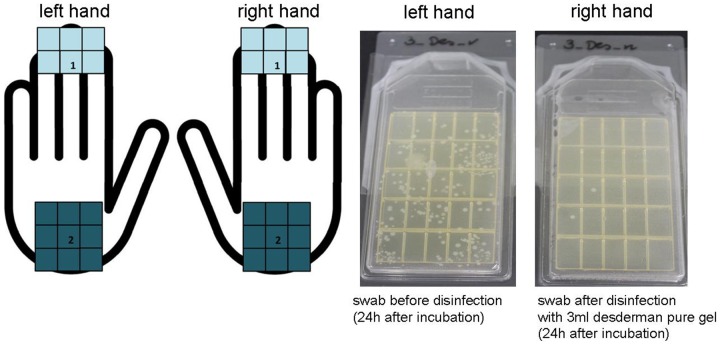
Standardized localisation for taking swabs of finger tips and hand palm before and after disinfection. (a) Agar plates with swabs of finger tips and hand palm before and after disinfection with desderman pure gel (phase 1); 24 h incubation at 35°C. (b).

One week later, phase two of the testing was conducted. The same 60 participants were briefed to travel to the University by public transport and also to contaminate their hands with everyday objects, in exactly the same manner as the previous week. Again, a swab of each subject's left palm and finger tips was taken and cultured on agar plates. The subjects were allocated to exactly the same hand sanitizer group as the previous week. However, in phase two the students were educated about correct hand disinfection procedures according to EN1500. [22] (Fig. 1) The key researchers of this study were intensively trained in EN1500 hand disinfection techniques before the project started. After receiving training in the correct use of hand rubs, the participants then applied their allocated hand rub utilising their recently acquired knowledge. A swab of their right palm and fingertips was taken and cultured on agar plates, incubated at 35°C for 24 hours and colonies were counted. (Fig. 3).

A statistical analysis of the counted colonies was performed, using mean, Student's t-test and standard deviation to evaluate the efficacy of each disinfectant, to compare the bacterial reduction achieved by each product and to evaluate any differences between the three products. As both EN1500 certified products had been previously evaluated, no significant difference was expected between the two and they were consequently considered as internal controls for the purpose of this study.

To compare the relative bacterial reduction after disinfection with the three different agents, the sum of all bacterial colonies before disinfection in each of the three groups was defined as 100%. The relative remaining bacterial burden was determined using the sum of bacterial colonies found in each group after disinfection.

After the first stage of the testing was completed, the 60 participants were asked to use all three hand disinfectants (hands were washed with plain water in between using each hand sanitizer), then complete a questionnaire about their perceptions of the three hand rubs and finally give scores to key attributes of hand rubs including efficacy, perfume, price and skin tolerability.

The practical part of this study was performed as a project in accordance with the University of Applied Science Campus Vienna, as exercises are required in current curriculum. Therefore the board of the University reviewed and approved the protocol. The project was officially supervised by two members of the University of Applied Science Campus Vienna.

All participants were healthy adult volunteers without documented health risks. Efficacy of study products was assessed without any additional or unnatural bacterial contamination. Volunteers were fully informed of study procedures and asked to give verbal consent prior to participation. According to European law no written consent is necessary and therefore was not obtained. The supervisors of the University approved this consent procedure.

Due to national - Austrian - as well as European laws an additional ethical committee approval was not appropriate since no medicine, drug or medical devices have been investigated. Tested products are commercial available hand disinfectants, intended to be used for hand disinfection and the aim of this study was to prove reduction of transient flora in laypersons by only a single application of one product. No additional dermatological parameters or any other clinical data were collected.

## Results

Before disinfection (in both phase one and two), there was no significant difference between the groups in the bacterial load detected on hands.

During phase one (before the volunteers received training in the optimum use of the hand rubs), there was a significant overall reduction in bacteria following hand disinfection with all three products. desderman pure gel, Sterillium and Lavit reduced the bacterial count to 6.4%, 8.2% and 28.0% respectively. These differences were significant (p<0.01) in bacterial reduction between desderman pure gel and Lavit as well as between Sterillium and Lavit.

**Figure 4:**
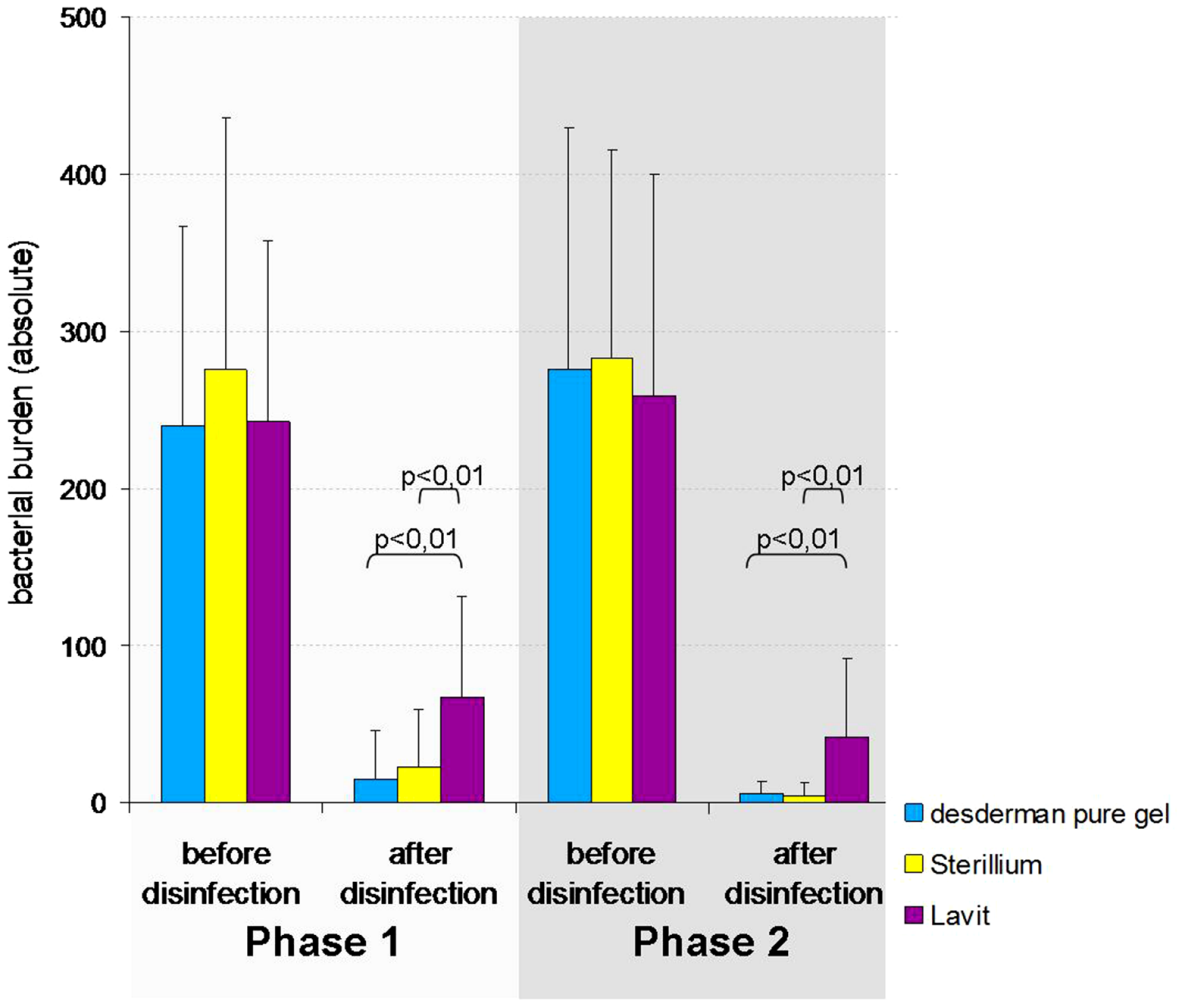
Bacterial burden in absolute numbers before and after disinfection (phase 1 =  without explanation of EN1500; phase 2 =  disinfection after explanation of EN1500). Standard deviation as indicated in the error bar.

Phase two of the study (after the participants were trained to EN1500 standards), again showed a significant bacterial reduction after disinfection with all three products. Disinfection with desderman pure gel, Sterillium and Lavit reduced bacterial burden to 2.1%, 1.5% and 16.2% respectively. As in phase one, these differences were significant (p<0.01) in bacterial reduction between desderman pure gel and Lavit as well as between Sterillium and Lavit. (Fig. 4).

**Figure 5:**
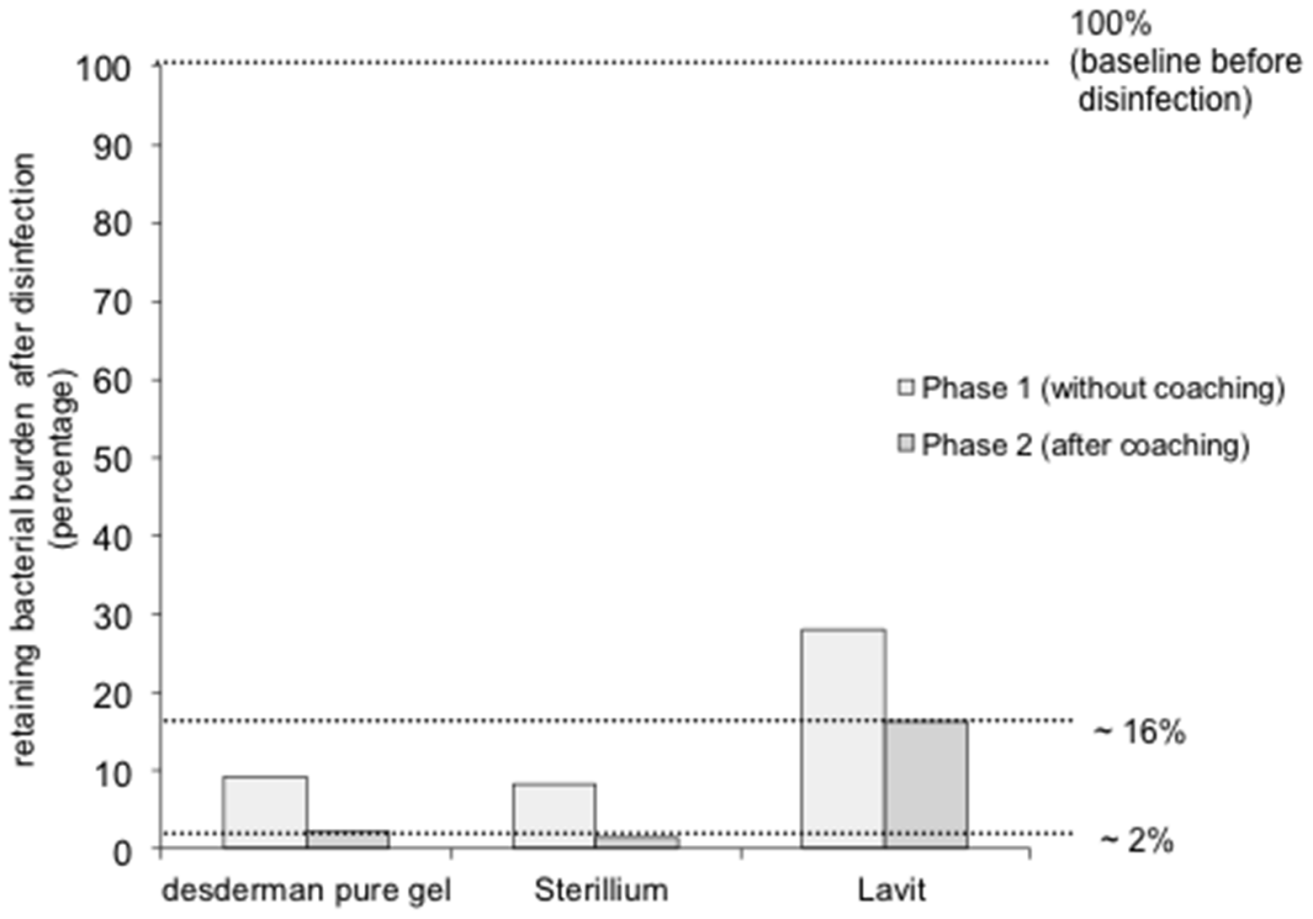
Relative remaining bacterial burden after disinfection. The sum of bacterial colonies in one group was taken as a baseline before disinfection (100%) and relative retaining bacterial burden was calculated with the sum of bacterial colonies of the same group after disinfection. After phase 1 (without explaining the EN1500 technique) relative retaining bacterial burden was 6.4%, 8.2% and 28.0% for desderman pure gel, Sterillium and Lavit, respectively. After phase 2 (disinfection according to EN1500) relative retaining bacterial burden was 2.1%, 1.5% and 16.2% when desderman pure gel, Sterillium or Lavit were used.

It was noted that when the subjects used Lavit according to EN1500 guidelines, the bacterial burden was still significantly higher (p<0.05) compared to the EN1500 certified products - desderman pure gel and Sterillium. Relative remaining bacterial burden after disinfection is shown in figure 5.

**Figure 6:**
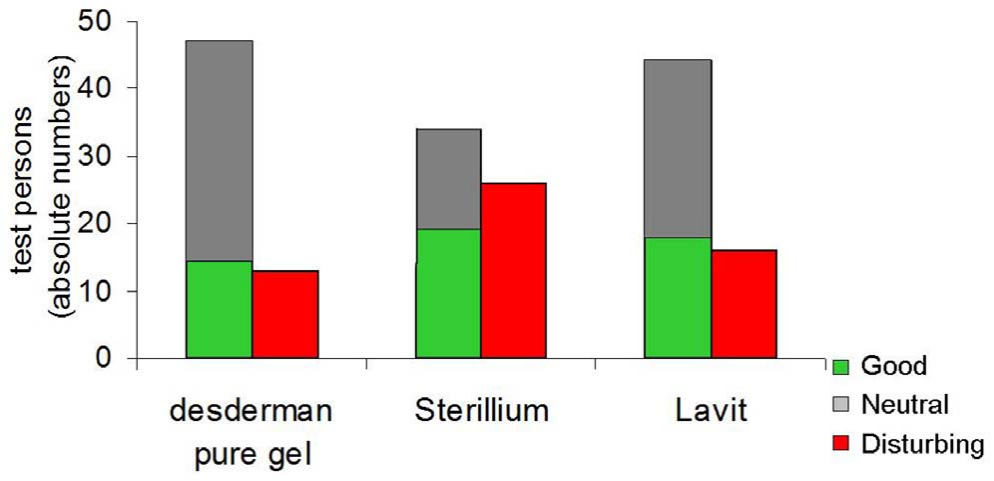
Number of participants that evaluated the odour of the three disinfectants as good (green), neutral (grey) or disturbing (red). The odour of desderman pure gel, Sterillium and Lavit were evaluated as good by 14, 19 and 18 persons, as neutral by 33, 15 and 26 persons and as disturbing by 13, 26 and 16 persons, respectively.

In terms of the completed questionnaires and the question of preferred scent, desderman pure gel was preferred; 78% (n = 47) either liked the odour or had no opinion. 73% (n = 44) and 57% (n = 34) of the test persons either liked or had no opinion concerning the perfume of Lavit and Sterillium, respectively. (Fig. 6) In terms of the perceived skin tolerability of each product, there was no significant difference between the three formulations, but there were differences in ‘ease of use’ perceptions; with ratings of 83%, 75% and 63% for Lavit, desderman pure gel and Sterillium respectively for being user-friendly. With respect to the attributes of a hand sanitizer, 43% rated efficacy as the most important priority, followed by skin compatibility by 34%, the cost of the product by 14% and the scent by 9%.

## Discussion

The use of both Sterillium and desderman pure gel led to a satisfactory bacterial reduction in both phases of the study. Lavit, which is not EN1500 certified, but is the most expensive product, did not reach comparable bacterial reduction in phase one or two. In phase one, the volunteers used on average four pumps of Lavit as there are no instructions from the manufacturer about the quantity of product to use. In phase two, 15 pumps of Lavit were consistently used, as well as the six steps of the EN1500 technique, but bacterial reduction was still significantly less than with Sterillium and desderman pure gel. 15 pumps of Lavit correspond to the 3 ml used of Sterillium and desderman pure gel. In practice, it is unlikely that 15 pumps or more would be used regularly for hand disinfection.

Although hand hygiene compliance in the general population has not been extensively studied, Judah *et al.* documented that one-quarter of adults in the general population show microorganisms of faecal origin on their hands, which implies low hand hygiene compliance after using the toilet. [24].

Regarding the efficacy of the three products, desderman pure gel (the only one containing ethanol) is claimed to be effective against a broad spectrum of viruses including norovirus, whereas the other two are not. This offers a potential advantage, as there is no vaccination against norovirus and outbreaks are regularly reported particularly on cruise ships, hotels, restaurants and public institutions.

A number of other public health-relevant viruses are only eliminated by using ethanol-based hand rubs including rotavirus (acute diarrhoea), rhinovirus (common cold), parvovirus (diarrhoea), hepatitis A-virus (liver infection) and adenovirus (conjunctivitis), respectively. The benefits of a hand rub containing ethanol were recently demonstrated by Cartner *et al.* [25], with the effects of three different alcohol based systems on the skin being investigated over two weeks. Alcohol based hand rubs containing n-propanol or isopropanol have demonstrated significantly greater skin irritation compared to ethanol-based ones. These results should be taken into account, when looking to improve compliance.

In 2002 Kramer *et al.* [26] investigated the antimicrobial efficacy of ten hand disinfectant gels, but none met the EN1500 requirements within 30 seconds of application. Therefore the authors concluded that alcohol-based gels should not replace liquid hand disinfectants in hospitals. Some years later the antibacterial efficacy and acceptability of an alcohol-based hand rinse compared with two alcohol-based hand gels was evaluated during routine patient care. Sterillium liquid showed comparable efficacy with Sterillium gel, but the overall hand hygiene compliance of health care workers improved when gel was available. [27].

In terms of skin compatibility and hand disinfectants, only occasional skin irritations have been reported on intact skin with the use of alcohol based hand rubs. The potential for skin irritation is reduced when moisturizers are added to the formulation of the hand rub. Where soap and water are used, reduced skin moisture is detectable. [28] In our study the odourless disinfectant was more popular than perfumed products and had the most preferred rating concerning odour. There is already a trend to supply fragrance-free hand hygiene products for healthcare professional use, which may be followed in products for the general population. This trend may be given further impetus as the role of fragrances in causing skin irritation is being investigated. [29].

When asked about skin compatibility in our study, there was no difference between the three products, but the results were different when asked about ease of use. The spray was rated as being the most user-friendly product. This may be due to the participants only using an average of four sprays in phase one, which was considerably less than the 3 ml used of Sterillium and desderman pure gel. It may also be due to the rapid evaporation experienced when using so little product. The liquid product was unpopular, because it dripped from the volunteers' hands, whilst this was not the case when using the gel or the spray.

Apart from differences in active ingredients, tested hand sanitizers contain different skin moisturizers.

Sterillium contains glycerol and Suchomel *et al.* demonstrated that glycerol significantly decreases the efficacy of alcohol based hand disinfectants in surgical application (three hour efficacy). [30] In the present study no significant differences in the potency of bacterial reduction between Sterillium and desderman pure gel were seen, but only short term efficacy was tested.

Isopropyl myristate is the moisturizing compound in desderman pure gel, acting as a skin moisturizer in very low concentrations. No negative reports about isopropyl myristate have been found in the literature.

Lavit contains Aloe Vera in an unknown concentration, but no other compounds are listed.

The results of our study should be of interest in the public sector, the leisure industry, institutions and any working environment which involves close contact with other people. Consideration should be given to making hand disinfectants readily available to all employees, this could be a cost effective measure if absenteeism through infectious illness is reduced. It has already been shown in preschool settings that routine hand hygiene measures deliver significant improvements in the reduction of infectious diseases. [31].

This study has some limitations. The efficacy of the disinfectants was only determined against bacteria, (although it is recognized that enveloped viruses like *Influenza* are eliminated by alcohol based hand rubs) and limited funding curtailed our ability to identify specific strains of bacteria. The correlation between the reduction in hand contamination and the reduction of infectious diseases was not investigated and would require a more complex study design.

Our sample size was small and further studies are recommended to explore our findings in greater depth. However, to the best of our knowledge this study is the first to evaluate the efficacy and ease of use of different formulations of hand rubs used by the general population.

## Conclusions

There are significant differences in efficacy between products that have been certified in accordance with the applicable European standards, compared to the non-certified product. The two certified products achieved superior outcomes compared to the non-certified product.

Furthermore, correctly performed hand disinfection according to EN1500 facilitates significant bacterial reduction, particularly when using EN1500 certified hand rubs.

Identifying the optimal methods to engage the general public with high standards of hand hygiene improvement is essential to facilitate behavioural change. Political commitment is essential to support campaigns aimed at increasing hand hygiene compliance. During influenza/norovirus outbreaks such promotion may contribute to saving lives in developed countries. It is universally recognized that hand hygiene is the best and most cost effective way to prevent infection and illness; and it is hoped that this study contributes unique information to the growing body of literature about hand hygiene.
